# Shikonin Causes an Apoptotic Effect on Human Kidney Cancer Cells through Ras/MAPK and PI3K/AKT Pathways

**DOI:** 10.3390/molecules28186725

**Published:** 2023-09-20

**Authors:** József Király, Erzsébet Szabó, Petra Fodor, Zsolt Fejes, Béla Nagy, Éva Juhász, Anna Vass, Mahua Choudhury, Gábor Kónya, Gábor Halmos, Zsuzsanna Szabó

**Affiliations:** 1 Department of Biopharmacy, Faculty of Pharmacy, University of Debrecen, 4032 Debrecen, Hungary; kiraly.jozsef@pharm.unideb.hu (J.K.); fodor.petra@pharm.unideb.hu (P.F.); vass.anna@pharm.unideb.hu (A.V.); konya.gabor@pharm.unideb.hu (G.K.); halmos.gabor@pharm.unideb.hu (G.H.); 2 Department of Pharmacology, Faculty of Pharmacy, University of Debrecen, 4032 Debrecen, Hungary; erzsebet.szabo@med.unideb.hu; 3 HUN-RE-DE Pharmamodul Research Group, University of Debrecen, 4032 Debrecen, Hungary; 4 Department of Laboratory Medicine, Faculty of Medicine, University of Debrecen, 4032 Debrecen, Hungary; fejes.zsolt@med.unideb.hu (Z.F.); nagy.bela@med.unideb.hu (B.N.J.); 5 Department of Pediatrics, Faculty of Medicine, University of Debrecen, 4032 Debrecen, Hungary; juhasze@med.unideb.hu; 6 Department of Pharmaceutical Sciences, Irma Lerma Rangel School of Pharmacy, Texas A&M Health Science Center, College Station, TX 77845, USA; mchoudhury@tamu.edu

**Keywords:** shikonin, human renal cancer, CAKI-2, A-498, apoptosis, Ras/MAPK and PI3K/AKT pathways, targeted therapy

## Abstract

(1) Background: Shikonin, the main ingredient in Chinese herbal medicine, is described as a novel angiogenesis inhibitor, and its anticancer effects have already been studied. Shikonin and its derivatives induce apoptosis and suppress metastasis, which further enhance the effectiveness of chemotherapy. However, their mechanism of function has not been completely elucidated on human renal cancer cells. (2) Methods: In our study, CAKI-2 and A-498 cells were treated with increasing concentrations (2.5–40 µM) of shikonin, when colony formation ability and cytotoxic activity were tested. The changes in the expression of the main targets of apoptotic pathways were measured by RT-qPCR and Western blot. The intracellular levels of miR-21 and miR-155 were quantified by RT-qPCR. (3) Results: Shikonin exerted a dose-dependent effect on the proliferation of the cell lines examined. In 5 µM concentration of shikonin in vitro elevated caspase-3 and -7 levels, the proteins of the Ras/MAPK and PI3K/AKT pathways were activated. However, no significant changes were detected in the miR-21 and miR-155 expressions. (4) Conclusions: Our findings indicated that shikonin causes apoptosis of renal cancer cells by activating the Ras/MAPK and PI3K/AKT pathways. These effects of shikonin on renal cancer cells may bear important potential therapeutic implications for the treatment of renal cancer.

## 1. Introduction

Despite recent advancements in screening and therapy techniques, renal cancer is still among the ten most common cancers worldwide. In Europe and North America, the lifetime risk for developing Renal Cell Carcinoma (RCC) ranges between 1.3% and 1.8%. According to the data provided by the World Health Organization, there are more than 140,000 RCC-related deaths yearly [1]. An improved understanding of biology has engendered novel targeted therapeutic agent development based on vascular endothelial growth factor, its related receptor, and the Mammalian Target of Rapamicyn (mTOR) signal transduction pathway [1,2,3]. Tyrosine kinase inhibitors, sunitinib, sorafenib, temsirolimus, and an immunoadjuvant, bevacizumab, have improved clinical outcomes in randomized trials [4,5]. However, there are newly discovered molecules that are effective in renal cancer therapy and may help overcome multidrug resistance (MDR), which is critical for successful therapy [6,7,8].

Shikonin (5,8-dihidroxy-2-((1*S*)-1-hydroxy-4-methylpent-3-en-1-y1)naphtalene-1,4-dione), a natural naphthoquinone compound, is isolated from the roots of the Chinese medical herb *Lithospermium erythrorhizon* [7,8]. These botanical sources can be found worldwide but the significant concentration is in Asia, as concluded from the literature available. Furthermore, the isolation of shikonin and its derivatives from these botanical sources was performed by chromatographic separation, the ultrasound-assisted method, and ultrasound-assisted ionic liquid solid-liquid extraction [9]. For thousands of years, shikonin has been widely used to treat sore throats, macular eruptions, measles, carbuncles, and burns [8,10]. Recently, emerging evidence has illustrated that shikonin has high efficacy against a series of human cancer cell lines in vitro and in vivo, with minimal toxicity to nonmalignant human cells [8,11,12,13,14]. Shikonin shows different anticancer activities, including the suppression of proliferation, inducing apoptosis in human lung adenocarcinoma cells, and attenuating invasion and migration in human breast cancer cells. Shikonin was reported to induce apoptosis, necrosis, or necroptosis in various cell lines, through signal regulation and molecular targets [7,8,13,15]. Also, shikonin is known to act on a variety of molecular targets associated with carcinogenesis and shows similar potency toward drug-sensitive and drug-resistant cancer cell lines [13,14]. Additional studies have revealed that shikonin exerts anticancer effects through the induction of apoptosis and autophagy in different cancer cells [8,14,16,17,18,19]. Shikonin-induced apoptosis was caused by the activation of the AKT-caused Apoptosis Signal-Regulating Kinase 1—p38 Mitogen-Activated Protein Kinase (AKT/ASK1/p38-MAPK) pathway and the downregulation of a cyclin-dependent kinase inhibitor (p21) in several cancer cells. Furthermore, it was reported that shikonin promotes apoptosis by activating the ER stress pathway and the mitochondrial apoptotic pathway in human prostate cancer cells [13,16,17,18].

Micro RNAs (miRNAs) are a group of endogenous noncoding small RNAs of 22–25 nts that regulate gene expression and are, therefore, involved in a variety of biological and pathological processes, including cellular differentiation, proliferation, apoptosis, and carcinogenesis. Certain studies have demonstrated the use of shikonin as a potential therapeutic agent to treat human glioblastoma by regulating miRNA expression profiles [11,15]. Recent evidence has indicated the role of miR-21 in the development and progression of human tumors, such as glioblastoma, hepatocellular, lung, colon, and prostate cancer [20]. Aimin et al. (2011) have shown that the reduction of miR-21 resulted in cell proliferation inhibition and cell apoptosis induction in renal cell carcinoma cells. Furthermore, miR-21 regulates multiple potential target genes and activates the caspase-3 pathway [20]. These raise the possibility of miR-21 as a potential therapeutic target for renal cell carcinoma, although more in-depth analysis is required [20].

In numerous human cancer types, miR-155 is overexpressed and demonstrates an oncogenic role. Previous studies have revealed that miR-155 has an important role in the progression of clear-cell renal cell carcinoma (ccRCC). It also takes part in the genesis of ccRCC by targeting Forkhead Box O3 (FOXO3a), a member of the RAS/MAPK signaling pathway, therefore becoming a potential target for ccRCC therapy [21].

Shikonin, as a potential therapeutic compound for renal carcinoma, has become an exciting and interesting topic; however, its effect and anticancer mechanism on kidney cancer cells have not been thoroughly investigated. Thus, this present study aimed to study whether shikonin alone could suppress the growth of human kidney cancer cells in vitro. One assumption is that some strong oncogenic miRNAs, such as miR-21 and miR-155, might have an epigenetic regulation on the process; therefore, we investigated the biological role of miR-21 and miR-155 on the underlying molecular mechanisms of shikonin on two human renal cancer cell lines in vitro. We also intended to describe the effect of shikonin on the genes involved in the apoptotic pathways leading to cancer cell death.

## 2. Results

### 2.1. Shikonin Inhibits Cell Proliferation in a Dose- and Time-Dependent Manner

**Figure 1:**
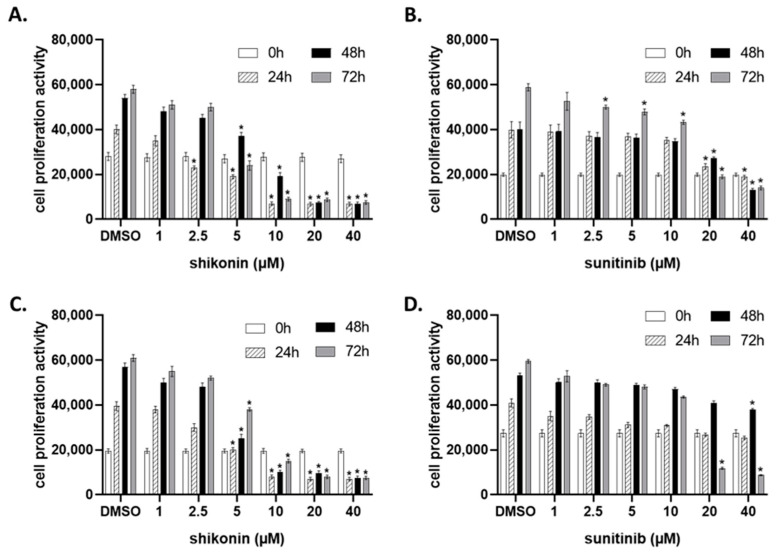
Cell proliferation activity of A-498 and CAKI-2 human renal cancer cells treated with shikonin or sunitinib: (**A**,**B**) A-498 and (**C**,**D**) CAKI-2 cells were plated into 96-well plates and treated with increasing doses of shikonin or sunitinib over 72 h. DMSO was used as a vehicle for nontreated control group. The cell proliferation activity was detected by Cell Titer Blue Assay. * *p* < 0.05—mean significant inhibition of cell growth.

In this study, two different clear renal carcinoma cell lines, A-498 and CAKI-2, were utilized to investigate the inhibitory effect of shikonin. To determine the effective dose of shikonin, the cells were incubated with increasing concentrations of the drug, and their cell proliferation ability was detected by the Cell Titer Blue Assay. The different concentration groups were compared with the Dimethyl Sulfoxide (DMSO) control group at different time points during a 72-h period. As shown in Figure 1, shikonin exhibited an antiproliferative effect on the A-498 and CAKI-2 cells and significantly suppressed cell proliferation in a dose-dependent manner (Figure 1A,C). To compare the effectiveness of shikonin with conventional drugs, the cells were treated with sunitinib (Figure 1B,D). The results showed that shikonin significantly suppressed the cell proliferation of both the A-498 and CAKI-2 cells as early as 24 h at a 2.5 µM concentration. However, the induced significant inhibition of cell proliferation in the A-498 cells by sunitinib was only observed at 20 µM (Figure 1B), and a detectable inhibition of CAKI-2 cell proliferation was observed at 40 µM only 48 h after the drug administration (Figure 1D). The inhibitory rate of shikonin gradually increased with time, indicating a time-dependent effect of shikonin on renal cell carcinoma cells. Morphological observation using an inverted microscope (Nikon Eclipse TS 100, Melvile, NY, USA) also showed an extremely pronounced effect of shikonin on both cell lines.

**Figure 2:**
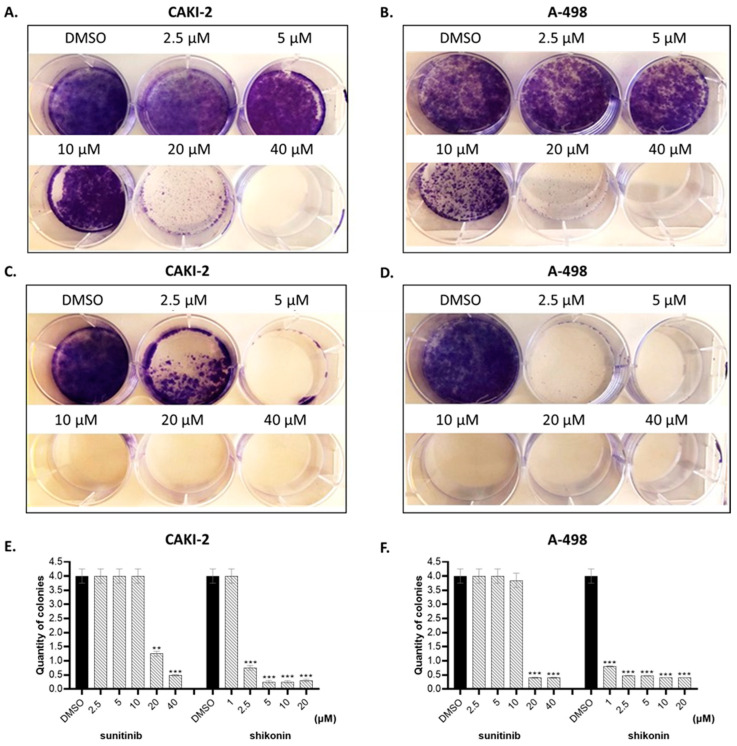
Inhibition of colony formation of CAKI-2 and A-498 human renal cancer cells by shikonin treatment. Cells plated into 6-well plates were treated with (**A**,**B**) sunitinib and (**C**,**D**) shikonin at the same time. After administration of the drugs, cells were cultivated for 14 days at 37 °C in a 5% CO_2_ incubator with 95% air. After termination of the experiment, the cells were fixed and visualized by 0.1% crystal violet solution. The colonies resuspended in 2% SDS solution were quantified by determination of absorbance at 570 nm. (**E**,**F**) Quantification of colonies formed by cells treated with sunitinib and shikonin in case of two different cell lines. **, *p* < 0.005; ***, *p* < 0.0005.

Next, we performed clonogenic cell survival assays to examine the inhibitory effect of shikonin on the clone formation ability of the chosen kidney cancer cells. Based on the proliferation activity assay results, the cells were treated with increasing doses of shikonin (1–20 μM) for two weeks for colony formation. We aimed to test and compare the effect of shikonin with sunitinib on the colony formation ability of CAKI-2 and A-498. As shown in Figure 2, shikonin effectively suppressed colony formation in both cell lines, CAKI-2 and A-498 (Figure 2C,D). Shikonin’s ability to inhibit colony formation proved to be more effective on the A-498 cells than on CAKI-2. There was a decrease of about three-fold in the number of colonies if 1 µM of shikonin was used in comparison to the control group in the A-498 cells and about eight-fold at the 2.5 µM concentration of the drug. In the CAKI-2 cells, the effect was pronounced at 2.5 µM (~six-fold) only. In accordance with the results revealed by the cell proliferation assay, shikonin suppressed the colony formation of kidney cancer cells at a much lower concentration (1 µM) than sunitinib. Sunitinib expressed the effect of a similar intensive inhibition on colony formation only at 20 µM (Figure 2).

These results suggest that shikonin may exhibit dose- and time-dependent inhibitory effects on the viability of kidney cancer cells (Figure 2). In addition, shikonin exerted a more pronounced inhibition on the cell proliferation of A-498 than on CAKI-2 cells.

### 2.2. Shikonin-Induced Apoptosis in Kidney Cancer Cells

**Figure 3:**
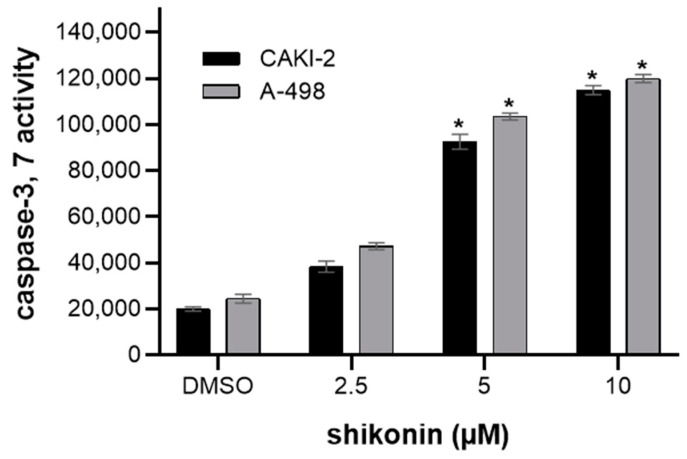
Effect of shikonin on apoptosis of CAKI-2 and A-498 human renal cancer cells. The cells were treated with shikonin and sunitinib over 48 h; the apoptosis was detected by measuring caspase-3 and -7 activity of the cells. * *p* < 0.05.

The induction of apoptosis by shikonin and its derivatives was demonstrated in many other cell lines. Caspases are considered to be critical modulators of apoptosis [22]. The activation of caspase-3 and -7 proteases is crucial in apoptotic cell death; therefore, the caspase-3 and -7 activity in shikonin-treated CAKI-2 and A-498 cells was determined. After 48 h, a ~six-fold increase in the caspase-3 and -7 activity over the control cells was observed in these cell lines. Caspase-3 and caspase-7 were significantly increased after treatment with shikonin, with the maximum activity peaking at 10 µM concentration (Figure 3).

**Figure 4:**
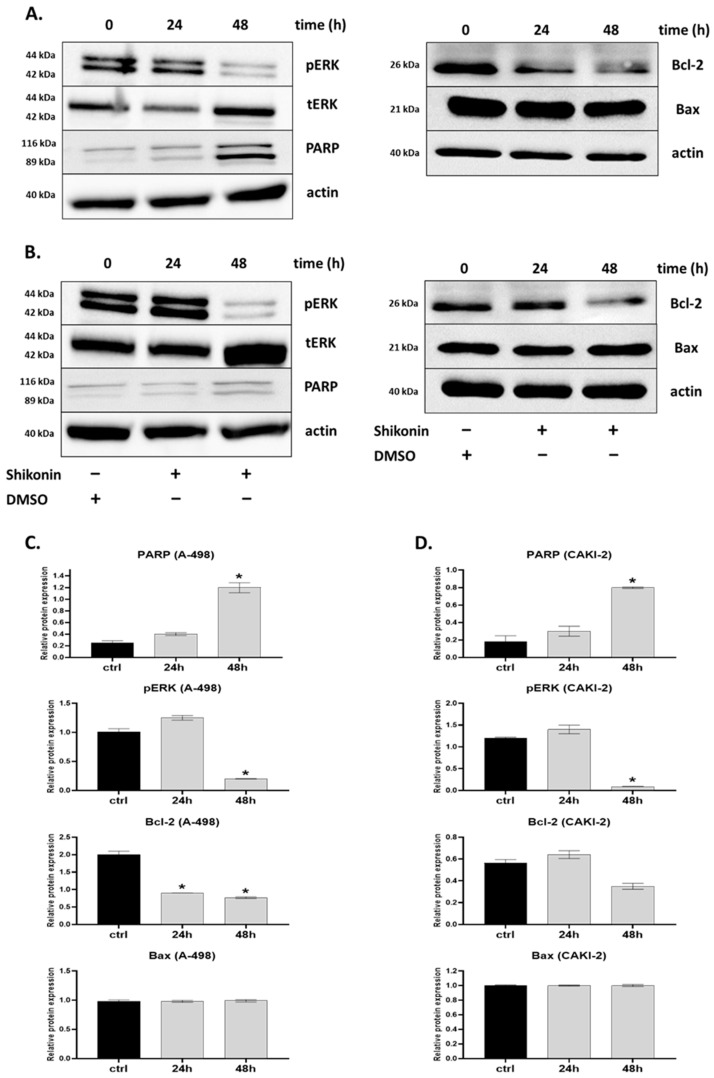
Western blot analysis of proteins associated with shikonin-induced cell apoptosis: A-498 and CAKI-2 human renal cancer cells were treated with 5 µM shikonin for 48 h. (**A**) The effect of shikonin on the expression of different proteins in A-498. (**B**) Expression level of proteins affected by shikonin treatment in CAKI-2 cells. (**C**,**D**) Bar graphs show the quantification of proteins normalized to β-actin or tERK (**C**—A-498 cells in the left-side column, **D**—CAKI-2 cells in the right-side column). Data were obtained from three independent experiments and were expressed as the mean ± S.D. Significant differences were calculated with two-way ANOVA analysis (* *p* < 0.05).

Since caspase-dependent apoptosis generally affects the cleavage of other proteins (e.g., poly (ADP-ribose) polymerase (PARP)), the expression of PARP after the shikonin treatment was investigated [18]. As demonstrated in Figure 4, the level of PARP expression was analyzed by immunoblotting and showed a significantly enhanced expression level of this protein 24 h after the treatment and a further increasing expression at 48 h. In the A-498 cells, the increase in PARP expression increased ~six-fold, and ~four-fold in the CAKI-2 cells over the untreated control group (Figure 4C,D).

The immunoblotting data proved that enhanced levels of caspase-3 and caspase-7 were accompanied by decreased levels of antiapoptotic protein, B-cell Lymphoma 2 (Bcl-2); however, for the expression level of the proapoptotic protein, Bcl-2 Associated X-protein (Bax), shikonin did not have a detectable effect in comparison to the untreated control cells (Figure 4A,B).

### 2.3. The Effect of Shikonin on the Expression of Apoptotic and Tumorsuppressor Genes

The Phosphatidylinositol 3-Kinase/AKT (PI3K/AKT) pathway is involved in fundamental cellular processes, including protein synthesis and cell proliferation. Also, the pathway plays a regulatory role in apoptosis. This pathway is activated by factors that induce PI3K, activating the mTOR pathways [23]. That is why we considered it important to study the changes in the pathway after shikonin treatment.

**Figure 5:**
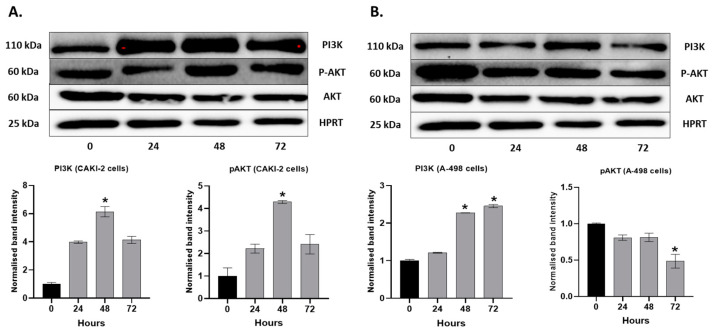
Western Blot analysis of the expression of apoptotic and tumorsuppressor proteins in human (**A**) CAKI-2 and (**B**) A-498 human renal cancer cell lines. The cells were treated with 5 µM shikonin for 24, 48, and 72 h, and total protein was isolated. A total of 40 µg of protein was applied to polyacrylamid gel, and the expressions of PI3K and pAKT were examined. The intensity of the protein bands was quantified using Image Lab software 5.2.1 (Bio-Rad Laboratories Inc., Hercules, CA, USA). The band intensity of shikonin-treated samples was normalized to untreated control samples. Data were obtained from three independent experiments and were expressed as the mean ± S.D. Significant differences were calculated with two-way ANOVA analysis (* *p* < 0.05).

In the human CAKI-2 (A) and A-498 (B) cell lines for PI3K and Phosphorylated-AKT (p-AKT), the protein expressions were also analyzed. The cells were treated with 5 µM shikonin during 24, 48, and 72 h periods. According to Western blot analyses, in the CAKI-2 cells, an increase in the expression level of PI3K and p-AKT was observed, reaching the highest at 48 h after treatment (Figure 5A). The A-498 cells showed an increasing expression throughout the experiment for PI3K, but a clear decrease was revealed in the p-AKT protein (Figure 5B).

Phosphatase and Tensin Homolog (PTEN) as a multifunctional tumor suppressor negatively regulates the intracellular levels of Phosphatidylinositol-3,4,5-Trisphosphate (PIP3) in the cells and negatively regulates the AKT/Protein Kinase B (AKT/PKB) signaling pathway. Also, it has been found to play an important role in shikonin-induced apoptosis [23].

**Figure 6:**
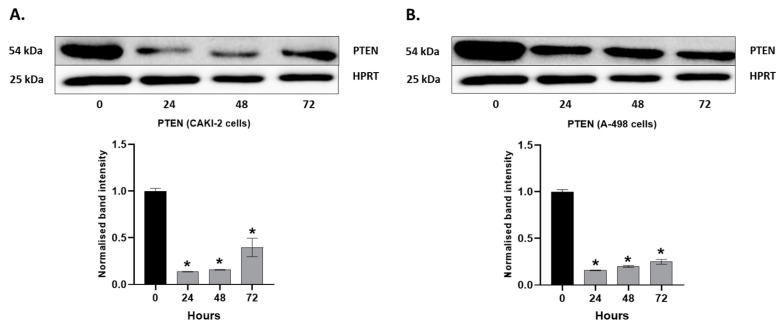
Western blot analysis for PTEN in human (**A**) CAKI-2 and (**B**) A-498 human renal cancer cell lines. The cells were treated with 5 µM shikonin for 24, 48, and 72 h. The intensity of the protein bands was quantified using Image Lab software (Bio-Rad Laboratories Inc.), and the signals were normalized to HPRT. The band intensity of shikonin-treated samples was normalized to untreated control samples. Data were obtained from three independent experiments and were expressed as the mean ± S.D. Significant differences were calculated with two-way ANOVA analysis (* *p* < 0.05).

A Western blot analysis was performed for the detection of PTEN at the protein level in human CAKI-2 and A-498 cell lines. The cells were treated with 5 µM shikonin for 24, 48, and 72 h periods. According to the results presented in Figure 6A,B for both cell lines, we observed a significant decrease in the expression of PTEN at the earlier stage, and then a slight increase over the treatment time.

### 2.4. MAPKs/PI3K Pathways Might Be Associated with Shikonin-Induced Cell Apoptosis

The mitogen-activated protein kinases (MAPKs) and PI3K/AKT pathways have key roles in regulating cell proliferation, cell survival, and apoptosis [23]. To evaluate shikonin’s capability of modulating the Extracellular signal-Regulated Kinases (ERK) signaling pathways, the effects of shikonin on the phosphorylation of p44/42 MAPK (tErk) were detected. As shown in Figure 4, the treatment of the A-498 and CAKI-2 cells with 2.5 µM shikonin for 24 h led to a significant inhibition of the Phospho—p44/42 MAPK (pErk)—expression level, in comparison to the expression levels of the total p44/42 MAPK protein. These results suggest that shikonin may downregulate the phosphorylation of these proteins in a time-dependent manner.

Collectively, the results indicate that shikonin inhibited the proliferation of kidney cancer cells through the induction of apoptotic cell death. Also, the results suggest that shikonin-induced apoptosis in kidney cancer cells could be mediated via a mitochondria-dependent pathway [22].

The Nuclear factor-kappaB (NF-κβ) is an inducible transcription factor that mediates signal transduction between the cytoplasm and nucleus in the cells. When NF-κβ is activated, it can upregulate the expression of key genes involved in the progression of cancer and the promotion of metastasis [24]. Several studies have reported about shikonin’s effect on the suppression of the DNA binding activity of NF-κβ [25]. This prompted us to investigate whether shikonin suppresses the expression of NF-κβ in A-498 and CAKI-2 cells.

**Figure 7:**
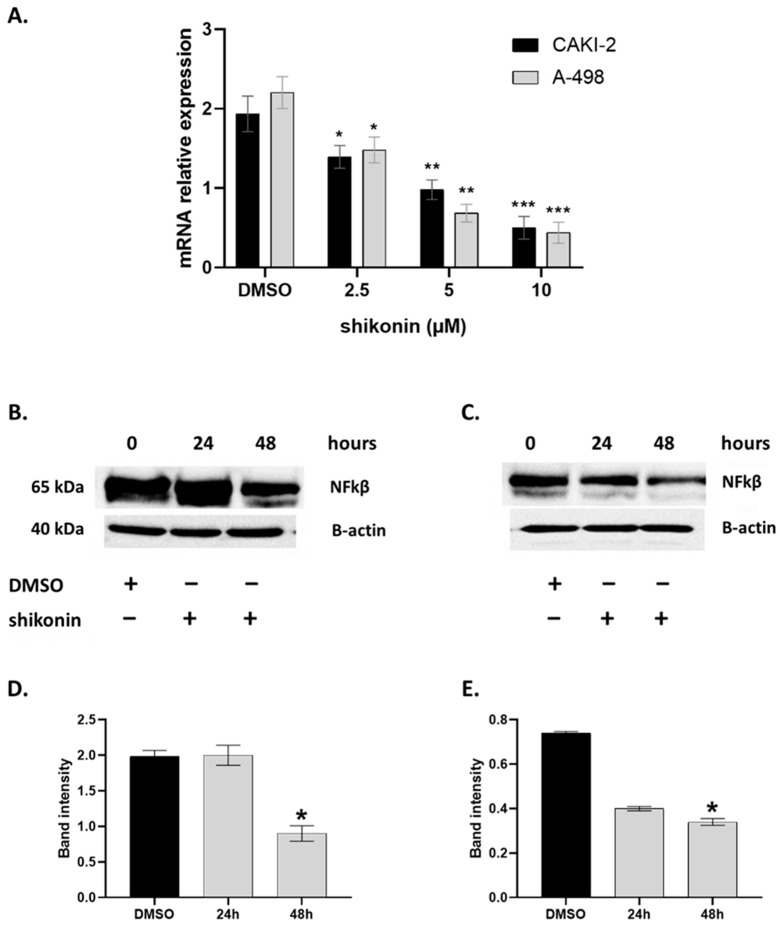
Shikonin inhibits the expression of NF-κβ. CAKI-2 and A-498 human renal cancer cells were seeded in 25 cm^2^ flasks. After 24 h of incubation, the medium was refreshed and the cells were treated with increasing doses of shikonin (2.5–10 μM) for 48 h. (**A**) mRNA expression of NF-κβ after shikonin treatment was quantified by qRT-PCR. (**B**) A-498 cells and (**C**) CAKI-2 cells represent level of NF-κβ expression at mRNA level in treated cells compared to nontreated control group (DMSO). (**D**,**E**) Quantified data of NF-κβ protein expression after shikonin treatment. Data were obtained from three independent experiments and were expressed as the mean ± S.D. * indicates a statistically significant difference compared to the DMSO nontreated control group: * *p* < 0.05, ** *p* < 0.005, and *** *p* < 0.0005.

Gene expression analyses of the NFkB gene in the CAKI-2 and A-498 cells after the treatment with increasing doses of shikonin (2.5–10 µM) within 24, 48, and 72 h incubation periods were performed by qRT-PCR, where Glyceraldehyde-3-Phosphate Dehydrogenase (GAPDH) served as the housekeeping gene. As it is shown in Figure 7, we observed a ~five-fold decrease in the NF-κβ messenger RNA (mRNA) expression in shikonin-treated A-498 and CAKI-2 cells compared to the nontreated control group. In accordance with the qRT-PCR data (Figure 7A), a significantly reduced level of the NF-κβ protein was detectable at 48 h (~two-fold) by Western blot analyses (Figure 7B–E).

Another aim of this study was to look for the relation between the NF-κB and the factors of apoptosis-like p53, which may affect tumor development and has a function in cell cycle control, drug resistance, and apoptosis, and also in the progression of frequently encountered adult renal tumors, ccRCCs [24].

**Figure 8:**
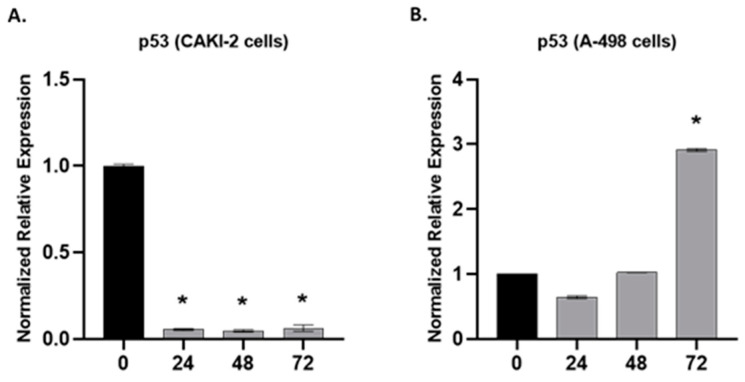
Gene expression analyses for p53 in human (**A**) CAKI-2 and (**B**) A-498 human renal cancer cell lines. The cells were treated with 5 µM shikonin; after 24, 48, and 72 h, total RNA was isolated from all of the samples. In qRT-PCR reaction, 40 ng of cDNA was used. GAPDH was used as a housekeeping gene. The expression levels of shikonin-treated samples were normalized to untreated control samples (* *p* < 0.05).

The expression of p53 at the mRNA level was studied in the CAKI-2 and A- 498 cells by qRT-PCR after the treatment of the cells with 5 µM shikonin for 24, 48, and 72 h. Figure 8A shows a significant decrease in the expression of p53 in the CAKI-2 cells (*p* < 0.05) immediately after 24 h of shikonin treatment. However, the A-498 cells revealed a slight decrease in the expression of p53 after 24 h of incubation with shikonin, while, after 72 h of treatment, we could observe a ~three-fold increase in the expression of the p53 gene, in comparison to the control cells (*p* < 0.05) (Figure 8B).

### 2.5. The Effect of Shikonin on the Expression of Multidrug Transporter Genes

Multidrug resistance (MDR) constitutes a unique and critical spectrum of drug resistance with serious therapeutic consequences. Overcoming MDR is still a challenge in cancer chemotherapy [26]. The overexpression of MDRs can confer cancer cells with multi-drug resistance, and the inhibition of tumor cell efflux can effectively increase the sensitivity of tumor cells to chemotherapeutic drugs [26].

**Figure 9:**
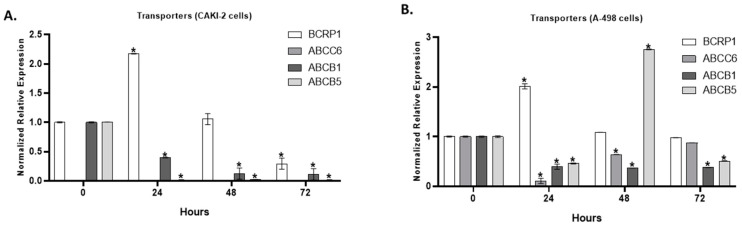
Expression of transporter genes in shikonin-treated (**A**) CAKI-2 and (**B**) A-498 human renal cancer cells. The cells were treated with 5 µM shikonin and collected after 24, 48, and 72 h of treatment, then total RNA was isolated. In qRT-PCR reaction, 40 ng of cDNA was used for the evaluation of the expression of BCRP1, ABCC6, ABCB1, and ABCB5 genes. GAPDH was used as a housekeeping gene. The expression levels of shikonin-treated samples were normalized to untreated control samples (* *p* < 0.05).

We aimed to investigate the expression of the most common multidrug transporter genes (Breast Cancer Resistance Protein 1 (BCRP1), ATP Binding Casette Subfamily C Member 6 (ABCC6), ATP Binding Casette Subfamily B Member 1 (ABCB1), ATP Binding Casette Subfamily C Member 5 (ABCB5)) of kidney cancer in the shikonin-treated A-498 and CAKI-2 cells. After 24 h of shikonin treatment, we observed a ~two-fold increase in the expression of the BCRP1 gene, both in the A-498 and CAKI-2 cells, which previously displayed a decreased expression in the control level after 72 h of shikonin treatment (Figure 9A,B). Significant differences (*p* < 0.05) in the expression of the BCRP1 gene were observed after 24 h of treatment in both cell lines. The expression of ABCC6 revealed a significant (*p* < 0.05) decrease after 24 h of incubation with shikonin in the A-498 cells, which increased to the control level after 72 h (Figure 9A,B). There is no expression of the ABCC6 transporter gene in the CAKI-2 cells, in either the control or the shikonin-treated cells. However, the ABCB1 expression significantly (*p* < 0.05) decreased in both of the cell lines, even directly after 24 h after treatment. The expression of ABCB5 almost completely decreased in the shikonin-treated CAKI-2 cells. The ABCB5 expression in the A-498 cells showed a 50% decrease after 24 h, which almost peaked up to a three-fold increase compared to the control untreated A-498 cells after 48 incubation hours with shikonin (Figure 9A,B).

### 2.6. The Effect of Shikonin on the Expression of the Extracellular Matrix Proteins

The steps of metastasis require the degradation of the extracellular matrix (ECM) constituents via proteolytic enzymes, including matrix metalloproteinases (MMPs), as the chief ECM-degrading enzymes [27]. As the expression and activation of MMPs are crucial for the degradation of ECM, which is necessary for cell invasion, we also examined the effects of shikonin on the Cadherins, C-X-C Motif Chemokine Receptor 4 (CXCR4), and MMPs expression.

**Figure 10:**
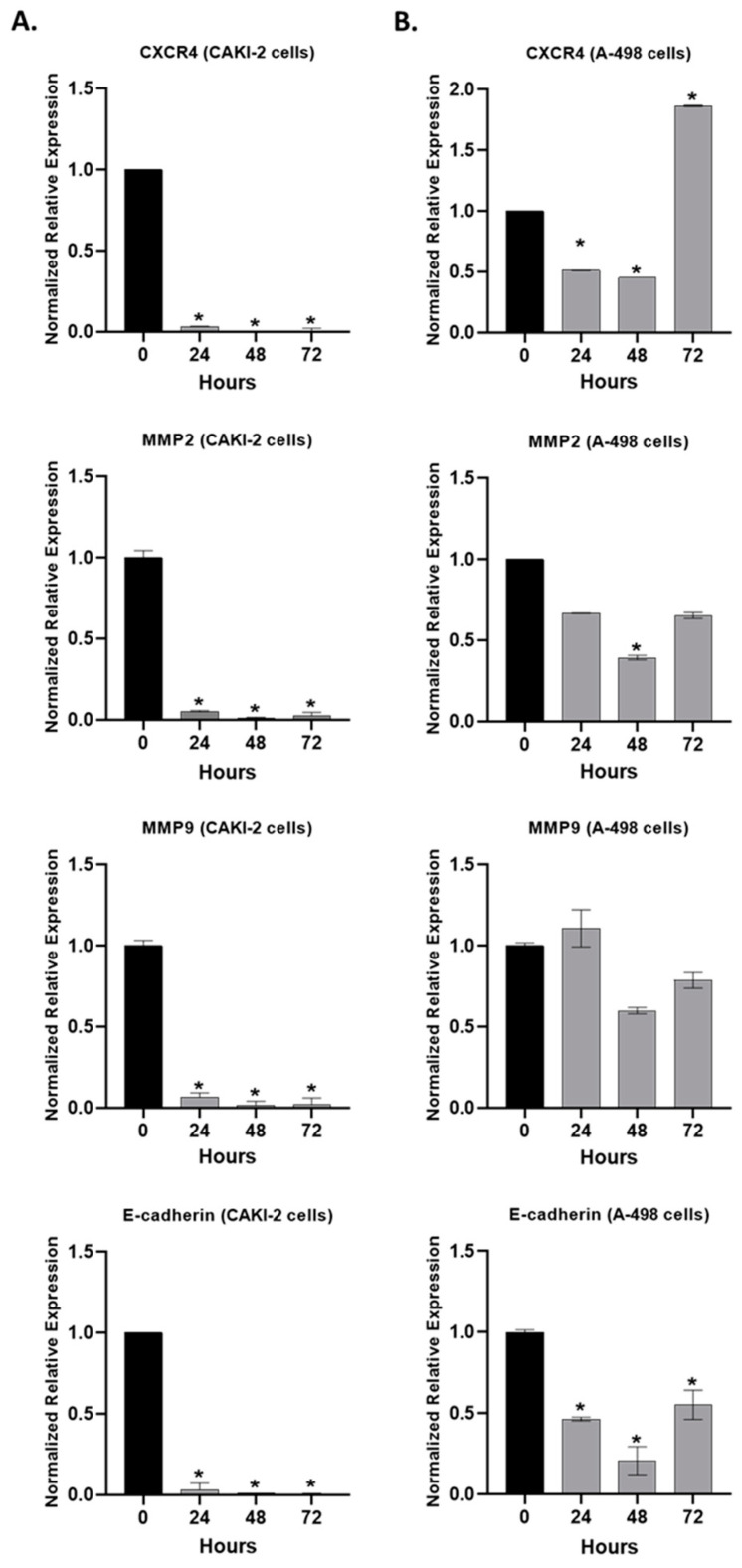
Expression of extracellular matrix genes in human (**A**) CAKI-2 and (**B**) A-498 human renal cancer cell lines after shikonin treatment. The cells were treated with 5 µM shikonin. After 24, 48, and 72 h, total RNA was isolated from all of the samples. A total of 40 ng of cDNA was used for gene expression analyses of CXCR4, MMP-2, MMP-9, and E-cadherin. GAPDH was used as a housekeeping gene (* *p* < 0.05).

The RT-qPCR results revealed that the shikonin treatment significantly (*p* < 0.05) decreased the expression of CXCR4, MMP-2, MMP-9, and E-cadherin in the CAKI-2 cells (Figure 10A). Also, a decrease in CXCR4, MMP-2, and E-cadherin in the A-498 cells (Figure 10B) was observed. However, in the expression of CXCR4, a ~two-fold increase can be seen after 72 h of treatment, compared to the untreated control cells. MMP-9 revealed a slight increase immediately after 24 h of treatment and then decreased after 48 h of incubation with shikonin (Figure 10B).

**Figure 11:**
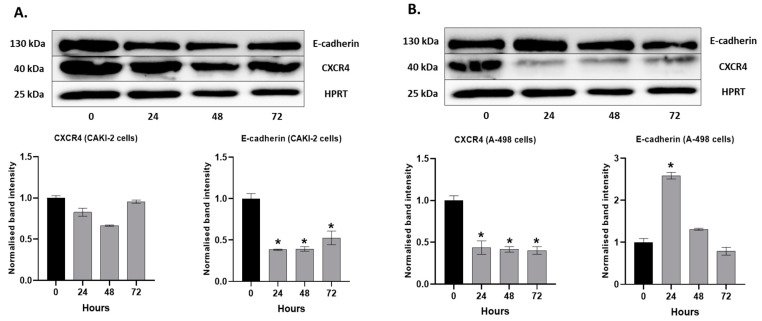
Western Blot analyses of the extracellular matrix protein expression in human (**A**) CAKI-2 and (**B**) A-498 human renal cancer cell lines. The cells were treated with 5 µM shikonin for 24, 48, and 72 h, and total protein was isolated. A total of 40 µg of protein was applied to polyacrylamid gel, and the expressions of E-cadherin and CXCR4 were examined. The intensity of the protein bands was quantified using Image Lab software (Bio-Rad Laboratories Inc.). The band intensity of shikonin-treated samples was normalized to untreated control samples. Data were obtained from three independent experiments and were expressed as the mean ± S.D. Significant differences were calculated with two-way ANOVA analysis (* *p* < 0.05).

The expressions of E-cadherin and CXCR4 were detected at the protein level as well. For the CAKI-2 cells, we observed a significant decrease in the expression of E-cadherin and a slight decrease in the expression of CXCR4. After 72 h of the treatment, we could see a sharp increase in the expression of both proteins compared to the 24 and 48 h samples (Figure 11A). In the A-498 cells, E-cadherin showed a significant increase in its expression after 24 h of shikonin treatment. However, after 48–72 h of incubation with shikonin, the expression of E-cadherin was decreased below the level measured in the control cells. The expression of CXCR4 significantly decreased after only 24 h following treatment, and there was no increase after the 48 and 72 h treatments (Figure 11B).

### 2.7. The Effect of Shikonin on the Expression of miR-21 and miR-155

MicroRNAs function as oncogenes or tumor suppressors and can regulate biological processes, including the cell cycle and apoptosis in tumor cell lines [28].

Both miR-155 and miR-21 serve as oncogenic miRNAs widely expressed in different human tissues and have been identified to be upregulated in numerous cancer types. They possibly control cell proliferation, migration, and invasion, as well as inhibit apoptosis [20,29,30].

**Figure 12:**
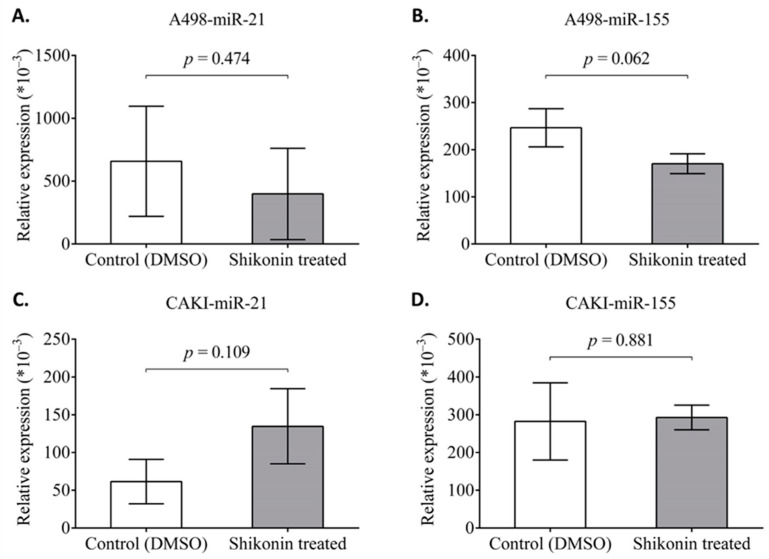
Effect of shikonin on miR-21 and miR-155 expressions in A-498 and CAKI-2 human renal cancer cells. CAKI-2 and A-498 cells were treated with shikonin for 48h. (**A**,**B**) Expression level of miRs was detected by qPCR in A-498 cells and (**C**,**D**) CAKI-2 cells.

Based on this background, we were additionally interested in miR-21 and miR-155 behavior in CAKI-2 and A-498 cells after shikonin treatment. The CAKI-2 and A-498 cells were incubated with shikonin for 48 h, the RNA was extracted, and an examination of the expression level of the miRNAs by qRT-PCR was completed. The results are presented in Figure 12. According to the data shown, there were no significant changes in the expression level of the examined miRs of the treated cells.

## 3. Discussion

Renal cell cancer (RCC) has an increasing incidence worldwide. Until recently, renal cell carcinoma has been a challenging disease to treat, due to limited therapeutic treatments [1,31]. A large group of patients suffer from preexisting resistance to conventional chemotherapeutic agents and radiotherapeutic regimens [1,2,3]. However, there are some newly discovered molecules that will be highly effective in the therapy of renal cancer. The efficacy of these novel agents creates a promising future for renal cancer therapy [2,3,6,7,8]. Several natural, low-toxicity plant extracts, including curcumin and shikonin, show an inhibition for a variety of cancer cell growth in vitro by inducing or suppressing the signaling pathways [18,32]. Some studies show that shikonin suppressed the proliferation of cancer cell lines (K562 cells) in a dose-dependent and time-dependent manner. Shikonin and its derivatives were shown to possess antiproliferative and proapoptotic effects in numerous tumor cells, including glioblastoma, sarcoma 180 (S-180), gastric cancer, colon adenomacarcinoma, and oral cancer cells [8,14,33,34].

Considering previous findings, this present investigation was conducted to evaluate the effect of shikonin on the induction of cell death and apoptosis in human renal cancer cells [19,35]. Shikonin showed a significant inhibition of cell proliferation at 2.5 µM in vitro. The drug exerted a dose-dependent inhibition of cell proliferation on both cell lines tested, and these results are in accordance with the records of the literature. According to the descriptions in the study of Markowitsch et al. (2022), the effect of the shikonin on proliferation was less pronounced than on cell growth, but it followed the dose-dependent course apparent for growth inhibition in therapy-sensitive (parental) and therapy-resistant CAKI-1, 786-O, KTCTL-26, and A498 RCC cells [36]. Shikonin administration also results in a dose-dependent proliferation reduction in prostate carcinoma cells [37]. Similarly, dose-dependent inhibition of proliferation by shikonin was evident in glioma cells and in the MCF-7 breast cancer cell line [12,36,38].

There was no previous evidence about the underlying mechanism of shikonin’s effect on cancer cell proliferation alone. Our aim with this present study was to investigate the underlying molecular mechanisms of only shikonin and its effects. For this purpose, we also analyzed the expression changes of the main multidrug transporters at the mRNA level. It is interesting to note that both BCRP1 and ABCC6 showed a significant decrease in the shikonin-treated A-498 cells immediately after the 24-h incubation. These results might suggest that shikonin enhances the antitumor effects by inhibiting the ABC transporter expression. The increasing expression of these MDR genes might contribute to the antitumor effect of some other drugs or could be an explanation for shikonin overcoming sunitinib-resistance of kidney cancer cells described by the work of Markowitsch et al. (2022) [36]. The deficiency of the ABCC6 transporter possibly has a genetic background in the CAKI-2 cells that may cause different behavior from A-498 on shikonin treatment and other therapeutical drugs. This ABCC6 activity in A-498 might be an explanation for the longer cell survival of shikonin-treated cells and also enhancing the apoptotic effects of the drug [39,40]. ABCB5 was found to have been decreased in both cell lines, as it strongly regulates a drug efflux that confers chemoresistance to human kidney cancer cells [41].

Shikonin suppresses the colony formation of kidney cancer cells at much lower concentrations than sunitinib. Previous studies have found that shikonin induces apoptotic cell death in a variety of cancers involving some specific multiple cellular targets [13,16]. Studies have also found in leukemia, bladder, and cervical cancer that shikonin could induce apoptosis by the activation of capase-3, which is similar to the findings of our study [19,35]. Shikonin inhibited the activity of topoisomerase II and NF-κβ, both of which are potential targets for chemotherapy. Furthermore, the antitumor activities of shikonin were mediated through the downregulation of antiapoptotic proteins, including Bcl-2 and Bcl-xL. This drug inhibited the action of Bcl-2, in turn releasing cytochrome-c, which leads to apoptosis by the activation of caspase-3 and caspase-7 [42]. Numerous other studies show that shikonin-induced apoptosis was associated with the activation of caspase-3 and PARP, which eventually results in apoptosis [18]. Previous studies proved caspase-3 activation and PARP cleavage in MCF-7 and HeLa cells support our results in A-498 and CAKI-2 cells [18,38].

In this current study, based on the results of the cell proliferation assay and by the measurement of caspase-3-activity, we showed that shikonin induced apoptosis in CAKI-2 and A-498 renal cancer cells in vitro. However, shikonin more strongly affects A-498 than the CAKI-2 cells. Significantly, shikonin enhanced its antitumor effect through the modulation of Nf-kβ - Bcl-2 and Nfk-linked genes and their products [15,17].

Protein P53 usually has a very low-level expression or is undetectable in cancer. It has a function in cell cycle control, drug resistance, and apoptosis. Wild-type p53 acts as a transcriptional regulator of the genes involved in cell cycle control; however, mutant p53 shows an advantage on the cells under hypoxic conditions, and tumor cells with mutant p53 are able to sustain a longer period of cellular proliferation [43]. A significant decrease in the expression of p53 was observed in CAKI-2 cells (*p* < 0.05) immediately after 24 h of shikonin treatment. However, the A-498 cells revealed a slight decrease in the expression of p53 after 24 h of incubation with shikonin, while, after 72 h of treatment, we could observe a ~three-fold increase in the expression of the p53 gene, compared to the control cells (*p* < 0.05). The changes in p53 in the CAKI-2 and A-498 cells after incubation with shikonin could explain that, in the PI3K/AKT pathway, AKT is regulating the expression of p53. PI3K mediates the activation of AKT via phosphorylation on Thr^308^ and Ser^473^ by Pyruvate Dehydrogenase Kinase 1 (PDK1) and PDK2, respectively. Protein P53 regulates the levels and status of the Bcl-2 family of proteins. The balance between the levels of the proapoptotic and antiapoptotic Bcl-2 family proteins controls the release of proapoptotic factors from the mitochondria. Once released, cytochrome-c activates firstly caspase-9 and then the executioner caspase-3, -6, and -7 [19,44].

One of the assumptions of the high decrease (five-fold decrease in NF-κB at mRNA level) in the expression of NF-κB in shikonin-treated A-498 and CAKI-2 cells compared to the nontreated control group can be that shikonin induces the Ras/MAPK and PI3K/AKT signaling pathways. In normal cells, NF-κB activation upregulates the transcription of its target genes. In tumoral cells, different types of molecular alterations may result in the impaired regulation of NF-κB activation. In some cases, NF-κB loses its inducibility and becomes constitutively activated, leading to the deregulated expression of the genes under NF-κB control [24].

Caspase-3 and -7 activities over the control cells were observed in the CAKI-2 and A-498 cell lines. Since caspase-dependent apoptosis is connected to the cleavage of PARP, we detected the expression level of PARP after the shikonin treatment. PARP showed a significantly enhanced expression level of protein at 24 h after the treatment and increased at 48 h. Similar results were reported by Soon Yang et al. (2014) [19]. Therefore, we can conclude that the antitumor activity of the shikonin exerted against CAKI-2 and A-498 human cancer cells is mediated through the downregulation of antiapoptotic proteins, including Bcl-2. The inhibition of Bcl-2 results in a release of cytochrome-c, and further leads to apoptosis by the activation of caspase-3 and -7. This might contribute to the proapoptotic activity of the p53 and NF-κβ genes. However, the inhibition of the NF-κβ mRNA expression revealed by qRT-PCR in the 48-h shikonin-treated A-498 and CAKI-2 cells indicates that the effect of the shikonin induces caspase-3 and -7 activation via the mitogenic protein kinase (MAPK) pathway, resulting in dose-dependent changes in the NF-ƙβ and Bcl-2 apoptotic genes [15,45]. We speculate that shikonin-induced apoptosis may also influence cell cycle redistribution through MAPK signaling [5].

To further elucidate the underlying mechanism, the effect of shikonin on the targets of the PTEN/PI3K/AKT signaling pathway was analyzed. Tyrosine kinase inhibitors (TKIs) are commonly used to treat kidney cancer; however, drug resistance and metastatic cases have limited the clinical application of TKIs. Shikonin might induce the PTEN/PI3K/AKT signaling pathway through their specific targets. Shikonin reduced the proliferation of the A-498 and CAKI-2 cells in a time- and dose-dependent manner and promoted the apoptosis of the cells. Moreover, shikonin increased the PI3K and p-AKT levels after 48 h of incubation intensively in CAKI-2 cells. The A-498 cells showed an increasing tendency for PI3K, but a clear decrease was revealed in the expression of the p-AKT proteins. According to the results, it is most likely that shikonin inhibits CAKI-2 and A-498 proliferation and migration by affecting the targets of the PTEN/PI3K/AKT pathway, revealing the possible effects of shikonin on the therapy of kidney cancer. A similar observation of the shikonin effect on the PTEN/PI3K/AKT pathway was observed in the study of Yu Chen et al. (2018) on CML cells [46].

PTEN is a multifunctional tumor suppressor that is commonly lost in human cancer. During 24, 48, and 72 h of incubation with 5 µM shikonin, the expression of the PTEN protein is significantly decreased in both the CAKI-2 and A-498 cell lines, and then a slight increase was revealed in the expression of PTEN, compared to the untreated cells. Western blot analyses proved our results at the protein level as well. PTEN likely has a negative effect on the regulation of the intracellular levels of phosphatidylinositol-3,4,5-trisphosphate in the cells and functions as a tumor suppressor by negatively regulating the AKT/PKB signaling pathway, thus playing a key role in shikonin-induced apoptosis. These results are in accordance with the Fan Ni et al. (2018) report, where PTEN has also been found to play an important role in shikonin-induced apoptosis [23].

The PTEN levels additionally inactivated the PI3K/AKT signaling pathway, subsequently upregulating Bcl-2 and Bax in the CAKI-2 and A-498 cells. The clear decrease in the extracellular matrix proteins, like CXCR4, MMP-9, and E-cadherin, suggests that in addition to an apoptotic and inhibiting effect, shikonin could likely block cell migration via the CXCR4/Stromal cell-Derived Factor 1 (SDF-1) axis. Consistently, shikonin significantly inhibits proliferation and promotes the apoptosis of kidney cancer cells in a very similar way to the cells isolated from Chronic Myeloid Leukemia (CML) patients [46].

A significant reduction in the expression levels of p-ERK, compared to the total protein expression levels of ERK after the 48-h treatment of the A-498 and CAKI-2 cells with 5 µM shikonin, suggests that shikonin may downregulate the phosphorylation of these proteins in a time-dependent manner [18,45].

The regulation of MAPK signaling, and the mitochondrial apoptotic pathways, may also involve some oncogenic miRNAs. MicroRNA-21 is involved in all the carcinogenesis phases, including initiation, promotion, progression, and metastasis. Studies have shown that upregulated miR-21 can induce Reactive Oxygen Species (ROS) production through the MAPK signaling pathway and is involved in the development of resistance to chemotherapeutic agents [47].

In breast, prostate, and hepatocellular carcinoma, it has been found that miR-21 controls migration and invasion by targeting PTEN [29]. Our results on miRNA suggest that MAPK is not a direct target of miR-21 in shikonin-induced apoptosis. Also, considering the expression pattern of miR-21 altogether with the expression of PTEN, we might conclude that miR-21 is likely not among the miRNA regulators that may cause migration and invasion by targeting PTEN [29].

miR-155 is one of the most characterized miRNAs and is a vital regulatory factor involved in multiple physiological processes, including hematopoietic lineage differentiation, immune response, and inflammation. miRNA profiling studies reveal that the level of miR-155 is upregulated in various types of human cancer and is associated with poor survival rates, therefore functioning as an oncogenic miRNA [30]. Though miR-155 is a strong oncogenic miRNA, miR-21 is not a direct target of the MAPK signaling pathway in the shikonin-induced pathological process. The relatively constant levels of miR-155 and miR-21 in shikonin-treated cells help us conclude that both CAKI-2 and A-498 cells have a strong genetically acquired drug resistance, which is possibly an explanation for the high TKI resistance behavior of the cell. This assumption was approved by some reports that discovered the close association of highly expressed miR-155 and acquired drug resistance in several human cancers, including breast cancer, B cell lymphoma, colon, lung, prostate, and cervical cancer [48].

In conclusion, we can say that shikonin induces both the PI3K/AKT and RAS/MAPK signaling pathways by regulating the specific targets’ signaling pathways, likely through the CXCR4/SDF-1 axis. The results of the miRNA expression analyses help us conclude that miR-21 and miR-155 have no significant role in the shikonin-effected pathway in the examined renal cancer cells and are not obvious epigenetic regulators of shikonin-induced apoptosis. However, other kidney cancer cell types may have a different reaction, and additional oncogenic miRNAs could be a part of the pathways shikonin is highly exertive in, thus creating additional ideas to be examined in further experiments. Intriguingly, many of the miRNAs that regulate apoptosis have been shown to affect cancer development. In the general process of the regulation of apoptosis, mainly miR-15b, miR-16, and miR-17b have been previously identified [49]. We also have to mention that the pathophysiology of renal cell carcinoma is mainly based on the von Hippel-Lindau/hypoxia-inducible factor 1-α (VHL/HIF) signaling pathway. Both the VHL and HIF1α genes were exhibited to be direct targets of miR-17-5p in RCC. This can be considered as one of the prominent miRNAs in the regulation of the process of apoptosis. In addition, miR-138 targets HIF1α and suppresses its expression, thus affecting apoptosis and the migration of ccRCC cells [50]. miR-708, also described as a tumor suppressor in RCC, plays an important role in cell growth, clonability, invasion, migration, and apoptosis [51].

Based on our results, we might suggest that shikonin possesses a strong antiproliferative and apoptotic effect. Our study substantially demonstrates that shikonin has the potential to be developed as an anticancerous and antiangiogenic drug in the therapy of renal cancer. Notably, shikonin exerted extremely high cytotoxicity in kidney cancer cells, but extremely low cytotoxicity in normal nasal epithelial cells [8,18]. In the future, shikonin and its derivatives can be considered an excellent drug candidate for the treatment of primary and metastatic renal cancers. It is also noteworthy that shikonin may provide a novel approach to overcome apoptosis-mediated drug resistance in human renal cancer cells. The use of (Arginyl-glycyl-aspartic acid: RGD) RGD-modified liposomes to deliver shikonin represents a potent strategy for therapy targeted against breast cancer [8]. This study suggests that shikonin combined with other chemotherapeutic agents might be an effective therapy for renal cancer [7].

## 4. Materials and Methods

### 4.1. Chemicals

Shikonin and sunitinib (purity > 98%) (Supplementary Figure S1A,B) were purchased from Santa Cruz Biotechnology (Dallas, TX, USA), dissolved in DMSO, and stored as a stock solution in aliquots at −20 °C. In the cell proliferation assay, the drugs were applied between 2.5 and 40 µM. DMSO was used as a vehicle in all experiments. Concentrations of the drugs used in further cell culture experiments were optimized according to the cell proliferation curves (Figure 1).

### 4.2. Cell Cultures

The A-498 and CAKI-2 human renal carcinoma cell lines were obtained from the American Type Culture Collection (ATCC) (Rockville, MD, USA). The cells were cultured in Iscove’s Modified Dulbecco’s Medium (IMDM) supplemented with 10% Fetal Bovine Serum (FBS) and antibiotics (100 U/mL penicillin and 100 μg/mL streptomycin). Both cell lines were maintained at 37 °C in a humidified atmosphere under 5% CO_2_/95% air. 

### 4.3. Detection of Cell Proliferation Activity

Cell Titer Blue Assay (Promega, Madison, WI, USA) was used for cell proliferation analysis. For the assay, A-498 and CAKI-2 cells (10^4^/well) were seeded in 96-well plates. All the cells were plated in their complete growth media 24 h before the experiments. The following day, the complete growth media was removed and replaced with the media containing increasing concentrations of the drugs (sunitinib or shikonin) for dose-response analysis. After the treatments, the cells were further incubated with the drugs over 72 h at 37 °C in the cell culture incubator with 5% CO_2_. The viability of the cells was detected every 24 h. The Cell-Titer Blue reagent (Promega) was added to the cells and, thus, the plates were incubated for 2 h at 37 °C in the cell culture incubator. Fluorescence intensity was measured using the BioTek Plate Reader system (BioTek, Winooski, VT, USA). All the data (cell proliferation activity) shown on Y-axis are a result of fluorescent activity detected at 560/590 nm.

### 4.4. Clonogenic Cell Survival Assay

Clonogenic Cell Survival Assay is a useful tool to test whether a given cancer therapy can reduce the clonogenic survival of tumor cells. The assay was performed on A-498 and CAKI-2 cells treated with sunitinib and shikonin. After the preparation of a single-cell culture suspension, 3–5 × 10^3^ cells/well were plated in six-well plates and seeded overnight. The next day, the cells were treated with different concentrations of shikonin (1–20 µM) or sunitinib (2.5–40 µM) (this range of concentrations was based on cell proliferation activity results). The control group of the cells was cultured in the complete cell culture media with 0.01% DMSO instead of the drugs. Afterward, the cells were allowed to grow for another 14 days. The media containing drugs was replaced with fresh media and drugs every third day. After 14 days, the experiment was terminated by removing the media. The cells were gently washed with Phosphate-Buffered Saline (PBS), fixed in methanol: acetic acid (3:1), and stained with 0.1% Crystal Violet solution (Sigma-Aldrich, St. Louis, MO, USA).

The photos were taken of each plate, and then, for quantification of the colonies, the cells were resuspended in 2% Sodium Dodecyl Sulfate (SDS) solution. The absorbance was measured by Biotek plate reader system (BioTek, Winooski, VT, USA).

### 4.5. Caspase-3 and -7 Activity Assay

Caspase-3 and caspase-7 activities were measured using the Caspase-Glo 3/7 assay (Promega, Madison, WI, USA) following the instructions outlined by the manufacturer. CAKI-2 and A-498 cells (10^4^) were plated into 96-well plates and treated with increasing concentrations of shikonin (2.5–10 μM). Forty-eight hours after the treatment, the reaction was completed and fresh media was added to each well of the cells. Afterward, the cells were lysed in 25 μL Caspase-Glow^®^ reagent and the plates were shaken for 30 min at room temperature (RT). Also, 100 μL of the lysate was transferred to a 96-well white wall plate and luminescence was measured on a BioTek plate reader (BioTek, Winooski, VT, USA).

### 4.6. Isolation of RNA and RT-PCR

To analyze the expression of NF-ĸβ, p53, CXCR4, MMP-2, MMP-9, E-cadherin, BCRP1, ABCC6, ABCB1, and ABCB5 at mRNA level, CAKI-2 and A-498 cells were treated with increasing doses of shikonin (2.5–10 µM) within 24, 48, and 72 h incubation periods, then total RNA was isolated. For total RNA extraction, NucleoSpin RNA/Protein commercial kit (Macherey-Nagel, Düren, Germany) was used.

### 4.7. Quantitative Real-Time PCR (qRT-PCR)

Quantities of mRNA for NF-ĸβ, p53, CXCR4, MMP-2, MMP-9, E-cadherin, BCRP1, ABCC6, ABCB1, ABCB55, and GAPDH (used as a housekeeping gene) were measured by the qRT-PCR method. A total of 40 ng of the earlier transcribed cDNA by RT-PCR was used for qRT-PCR, carried out with gene-specific primers (Supplementary Table S2). Real-time PCR was performed with iTaq™ Universal SYBR^®^ Green Supermix using a CFX-96 Real-Time System (BioRad Laboratories Inc., Hercules, CA, USA) in 20 µL final reaction volume. The reaction mixture was preheated at 95 °C for 10 min, followed by 45 cycles at 95 °C for 15 s and 60 °C for 1 min. The mRNA relative value was measured by ∆∆Ct method with threshold cycle times of the target genes and GAPDH.

### 4.8. Western Blot Detection of Proteins

The cells were treated with 5 μM shikonin for 24, 48, and 72 h. After the treatment, the cells were washed with PBS and lysed in protein lysis buffer (M-PER, Thermo Fisher Scientific, Waltham, MA, USA), containing protease and phosphatase inhibitors (Sigma-Aldrich, St. Louis, MO, USA). Protein quantification of cell lysate was performed using Bicinchoninic Acid (BCA) reagent (Thermo Fisher Scientific, Waltham, MA, USA). Samples were diluted with 4× Laemmli buffer and boiled at 95 °C for 8 min. Equal volumes (40 μg) were loaded on 10% or 14% sodium dodecyl sulfate–polyacrylamide gel and run by electrophoresis (SDS-PAGE). The protein lysates were separated corresponding to the molecular weight. As a molecular weight marker, Precision Plus Protein Kaleidoscope Standard (BioRad Laboratories Inc., Hercules, CA, USA) was used. The proteins from the gels were transferred to a polyvinylidene fluoride (PVDF) membrane (Millipore, Burlington, MA, USA). The blots were probed with specific antibodies, listed in Supplementary Table S1. Incubation with primary antibodies was followed by horseradish peroxidase (HRP)-tagged anti-mouse IgG or anti-rabbit IgG secondary antibodies (Thermo Fisher Scientific, Waltham, MA, USA). The signal was detected by chemiluminescence technique. For each sample, band intensities were normalized to beta-actin or Hypoxanthine Phosphoribosyltransferase (HPRT) (Sigma-Aldrich, St. Lous, MO, USA) or total ERK in the case of pERK (Cell Signalling, Danvers, MA, USA).

### 4.9. MicroRNA (miRNA) Specific Stem-Loop RT-qPCR Analysis

To perform miRNA analyses, CAKI-2 and A-498 cells were treated with increasing doses of shikonin (2.5–10 µM) within 24 and 48 h incubation periods, then total RNA was isolated (Macherey-Nagel, Düren, Germany). The expression of intracellular miRNAs, miR-21 and miR-155, were quantified by miRNA-specific Universal ProbeLibrary (UPL)-probe-based stem-loop RT-qPCR method as recently described in [52,53].

Primers and qPCR assays were designed using the software developed by Czimmerer et al. (2013) and oligonucleotides used in this study are listed in Supplementary Table S3 [53,54]. This quantification technique included two steps:

(1) miRNAs transcription into cDNA via reverse transcription was performed from total RNA (10 ng) using miRNA-specific stem loop-RT primer (500 nM, Integrated DNA Technologies, Leuven, Belgium) and TaqMan^®^ MicroRNA Reverse Transcription Kit (Applied Biosystems, Foster City, CA, USA).
(2) miRNA quantification was performed by RT-qPCR using designed miRNA-specific forward primer (100 μM, Integrated DNA Technologies), universal reverse primer (100 μM, Integrated DNA Technologies), and UPL probe #21 (10 μM, Roche Diagnostics, Mannheim, Germany) with Taq polymerase (5 U/μL) and dNTPs (2.5 mM) (Thermo Scientific, Wilmington, DE, USA).

The RT-PCRs were carried out in triplicates on a QuantStudio 12 K Flex qPCR instrument (Applied Biosystems). The final reaction volume (10 μL) was preincubated at 95 °C for 1 min and was followed by 40 cycles of 95 °C for 15 s and 60 °C for 30 s. For normalization, the small-nucleolar RNU-43 was measured as a reference gene using the aforementioned method [52,53].

### 4.10. Statistical Analysis

All experiments were performed at least three times. The evaluation and generation of mean values, the associated standard deviation, and normalization in percent were performed by Microsoft Excel (Office Professional Plus 2016, Microsoft, Redmond, WA, USA). Statistical significances were calculated with GraphPad Prism 5.0 (GraphPad Software Inc., San Diego, CA, USA) with two-way ANOVA test. Differences were considered statistically significant at a *p*-value ≤ 0.05.

## 5. Conclusions

Shikonin showed a dose- and time-dependent inhibitory activity on kidney cancer cell lines. Our results suggest that shikonin may cause an apoptotic effect through the target proteins of Ras/MAPK and PI3K/AKT pathways, without the direct epigenetic regulators of the examined miRNAs, such as miR-21 and miR-155. However, the direct involvement of other miRNAs should be considered. Hopefully, in the future, further in vivo experiments will prove our concept that shikonin could become a potential drug candidate for the treatment of human kidney cancers.

## Data Availability

The data used to support the findings of this study are included within the article.

## Supplementary Materials

The following supporting information can be downloaded at https://www.mdpi.com/article/10.3390/molecules28186725/s1, Figure S1: Chemical structure of drugs used in the experimental work; Table S1: List of the antibodies used for Western blots, Table S2: List of the primer sequences used for qRT-PCR, Table S3: miRNA sequences and primers for reverse transcription.
