# Supplementary material for: Shikonin Causes an Apoptotic Effect on Human Kidney Cancer Cells through Ras/MAPK and PI3K/AKT Pathways
Source: Molecules. 2023 Sep 20;28(18):6725. doi: 10.3390/molecules28186725 (PMC10534756; doi:10.3390/molecules28186725)
Supplement: Supplementary file 1 [file molecules-28-06725-s001.zip › molecules-2597852-supplementary.pdf]

A. **B.**

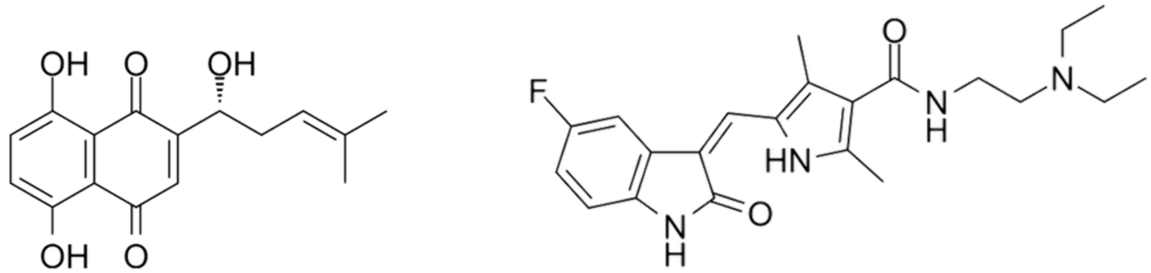

Figure S1: Chemical structure of drugs used in the experimental work. (**A**) Shikonin and (**B**) Sunitinib (www.medchemexpress.com)

Table S1: List of the antibodies used for Western blots

Antibody	Origin, catalog number	Dilution applied
PARP (46D11) Rabbit mAb	Cell Signalling, #9665	1:1000
NF-kappaB p65 (D14E12) XP Rabbit mAB	Cell Signalling, #8242	1:1000
Bcl-2(D5568) rabbit mAb	Cell Signalling, #4223	1:1000
Bax (D2E11) rabbit mAb	Cell Signalling, #5023	1:1000
Phospho-p44/42MAPK (ERK1/2) Thr202/Tyr 204 Rabbit mAB	Cell Signalling, #9101	1:1000
p44/42MAPK (ERK1/2) Thr202/Tyr 204 Rabbit mAB	Cell Signalling, #9102	1:1000
AKT (pan) (11E7) Rabbit mAB	Cell Signaling #4685S	1:1000
Phospho-AKT (S473) (D9E) Rabbit mAB	Cell Signaling #4060S	1:1000
PI3K p110alpha (C73F8) Rabbit mAB	Cell Signaling #4249S	1:1000
PTEN (D4.3) XP(R) Rabbit mAB	Cell Signaling #9188L	1:1000

CXCR4 (771) Rabbit mAB	Affinity Biosciences #AF5279	1:1000
Anti-HPRT HPRT1 (P00492) Rabbit mAB	BOSTER Biological Technology #M00668	1:2000
Monoclonal anti β-actin produced in mouse	Sigma #A5316	1:2000

Table S2: List of the primer sequences used for qRT-PCR.

Primer	Forward	Reverse
NF-κβ	5′-GCAAAGGGAACATTCCGATAT-3′	5′-GCGACTCACATGGAAATCTA-3′
p53	5′-CCTCAGCATCTTATCCTAGTGG-3′	5′-TGGATGGTGGTACAGTCAGAGC-3′
MMP-2	5′-TACTGGATCTACTCAGCCAGCA-3′	5′-CTTCAGGTAATAGGCACCCTTG-3′
MMP-9	5′-GGGCTTAGATCATTCCTCAGTG-3′	5′-GCCATTCACGTCGTCCTTAT-3′
E-cadherin	5′-GAAAGCGGCTGATACTGACC-3′	5′-CGTACATGTCAGCCGCTTC-3′
BCRP1	5′-GTTCTCAGCAGCTCTTCGGCTT-3′	5′-TCCTCCAGACACACCACGGATA-3′
ABCC6	5′-AGGCTTTCCTGCCCTTCCCCAT-3′	5′-CCAGAGGAACTTGAGTCTACGAC-3′
ABCB1	5′-GCTGTCAAGGAAGCCAATGCCT-3′	5′-TGCAATGGCGATCCTCTGCTTC-3′
ABCB5	5′-TTTGCCTATGCGGCAGGGTTTC-3′	5′-CAAAACGAGCGTTTCTCCGATGG-3′
GAPDH	5′-TGTAGTTGAGGTCAATGAAGGG-3′	5′-ACATCGCTCAGACACCATG-3′

Table S3: miRNA sequences and primers for reverse transcription.

miRNA names	Mature miRNA sequences	Stem-loopprimers for reverse transcription
hsa-miR-21-5p	5′ - UAGCUUAUCAGACUGAUGUUGA - 3′	5' - GTTGGCTCTGGTGCAGGGTCCGAGGTATTCGCACCAGAGCCAAC TCAACA - 3'
hsa-miR-155-5p	5′ - UUAAUGCUAAUCGUGAUAGGGGU - 3′	5' - GTTGGCTCTGGTGCAGGGTCCGAGGTATTCGCACCAGAGCCAAC ACCCCT - 3'
hsa-RNU-43	5' - GAACUUAUUGACGGGCGGACAGAAACUGUGUGCUGAUUGUCACGUUCUGAUU - 3'	5' - GTTGGCTCTGGTGCAGGGTCCGAGGTATTCGCACCAGAGCCAAC AATCAG - 3'
